# Next generation methodology for updating HA vaccines against emerging human seasonal influenza A(H3N2) viruses

**DOI:** 10.1038/s41598-020-79590-7

**Published:** 2021-03-02

**Authors:** James D. Allen, Ted M. Ross

**Affiliations:** 1 Center for Vaccines and Immunology, University of Georgia, 501 D.W. Brooks Drive, CVI Room 1504, Athens, GA 30602 USA; 2 Department of Infectious Diseases, University of Georgia, Athens, GA USA

**Keywords:** Immunology, Microbiology

## Abstract

While vaccines remain the best tool for preventing influenza virus infections, they have demonstrated low to moderate effectiveness in recent years. Seasonal influenza vaccines typically consist of wild-type influenza A and B viruses that are limited in their ability to elicit protective immune responses against co-circulating influenza virus variant strains. Improved influenza virus vaccines need to elicit protective immune responses against multiple influenza virus drift variants within each season. Broadly reactive vaccine candidates potentially provide a solution to this problem, but their efficacy may begin to wane as influenza viruses naturally mutate through processes that mediates drift. Thus, it is necessary to develop a method that commercial vaccine manufacturers can use to update broadly reactive vaccine antigens to better protect against future and currently circulating viral variants. Building upon the COBRA technology, nine next-generation H3N2 influenza hemagglutinin (HA) vaccines were designed using a next generation algorithm and design methodology. These next-generation broadly reactive COBRA H3 HA vaccines were superior to wild-type HA vaccines at eliciting antibodies with high HAI activity against a panel of historical and co-circulating H3N2 influenza viruses isolated over the last 15 years, as well as the ability to neutralize future emerging H3N2 isolates.

## Introduction

Influenza A viruses (*Orthomyxoviridae*) from the A(H1N1)pdm09 and A(H3N2) subtypes currently circulate in humans and cause severe illness in 3–5 million people annually. The World Health Organization (WHO) estimates that these severe infections result in 290,000 to 650,000 respiratory related deaths every year^1,2^. In general, since 1968 when A(H3N2) viruses emerged in the human population, influenza seasons in which A(H3N2) viruses are prevalent, tend to be more severe with a greater number of hospitalizations and deaths^3^. The 2017–2018 influenza season, dominated by A(H3N2) virus circulation, was particularly severe and led to the third most outpatient visits for influenza virus-like illnesses in the last 20 years, causing over 900,000 hospitalizations and 80,000 deaths^4,5^. Influenza virus vaccine effectiveness estimates vary across seasons and between different age groups, but they are typically higher against influenza A(H1N1)pdm09 and influenza B than they are against A(H3N2) viruses^6^. During the 2018–2019 influenza season, the A(H3N2) component elicited immune responses that only protected individuals against ~ 9% of A(H3N2) virus infections^7,8^.

Despite low vaccine effectiveness, vaccination remains the cornerstone to prevent influenza virus infection^9^. Annual seasonal influenza virus vaccines in the United States are typically composed of two influenza A strains representing the A(H1N1) and A(H3N2) subtypes and either one or two influenza B strains representing the Victoria and Yamagata lineages^10,11^. In some countries trivalent forms of the vaccine containing one H1N1, H3N2, and influenza B strain are still used. Strains from these Influenza A and Influenza B subtypes currently co-circulate and pose a threat to human health and well-being^12^. In particular, influenza A(H3N2) viruses are associated throughout recent history with widespread influenza virus induced illness^13–16^.

Since their introduction to the human population in 1968, A(H3N2) influenza viruses have undergone extensive genetic drift and antigenic evolution leading to numerous seasonal epidemics, exemplified by the WHO recommending 29 A(H3N2) vaccine strain changes over the last 50 years^17^. The rapid evolution of influenza A(H3N2) viruses creates difficulties for experts to recognize and predict current and future epidemiological threats^5,16,17^. Typically, the strains that are chosen for use in the vaccine are selected based on predictions of predominant or emerging clades of influenza viruses derived from global surveillance information, but this method can often lead to the selection of antigenically mismatched strains that induce limited antibody protection against co-circulating viral variants^1,8^. Antigenic mismatches between the chosen vaccine strain and currently circulating viruses often lead to reduced vaccine effectiveness, requiring influenza vaccines to be updated frequently^18^.

Designing an influenza virus vaccine that induces both broad antibody reactivity against co-circulating strains and neutralization across multiple future influenza virus seasons is a pivotal challenge for the development of new influenza vaccines^19^. In order to address the need for more broadly reactive influenza A vaccines, a previously reported methodology for enhanced antigen design, computationally optimized broadly reactive antigen (COBRA), which utilizes multiple rounds of layered consensus building to generate influenza vaccine HA immunogens for H1, H3, and H5 influenza subtypes has shown great promise^19–26^. COBRA HA antigens are capable of eliciting broadly reactive HA-specific antibody responses that can protect against both seasonal and pandemic influenza strains that have undergone genetic drift^19,23,24^. These vaccine antigens have also been shown to inhibit viral infection and virus induced pathogenesis in various animal models including mice, ferrets, and non-human primates^22,27–29^.

Adapting a broadly reactive influenza virus vaccine design technology, such as COBRA, to industrial settings would be extremely beneficial for both manufacturers and consumers. Aside from potentially reducing the need to update vaccines on an annual basis, technologies like the COBRA methodology provide a promising solution to increase the protection offered by seasonal vaccines against currently co-circulating strains as well as future emerging isolates. The current vaccine manufacturing process takes about 6–8 months from strain selection to vaccine availability and any delays in strain recommendation or antigenic characterization can lead to devastating reductions in the vaccine supply^1,30^. Thus, having a broadly reactive vaccine candidate, that has already been antigenically characterized, optimized for production, and is “shelf-ready” would save manufacturers a considerable amount of stress and time, while allowing for year-round production of more doses of vaccine.

Previous COBRA design methodologies focused on developing antigens that are broadly reactive against historical and contemporary influenza vaccine strains^22,24,26^. However, since A(H3N2) viruses evolve so rapidly through genetic mutation and reassortment, it is likely that the protection offered by a broadly reactive vaccine candidate designed using historical isolates, antigenic eras, and outbreak groups will eventually begin to decline due to the diversity of newly emerging surface epitopes present in the circulating A(H3N2) viral population^24,31^ . Therefore, there is a need to develop a method for updating these broadly reactive vaccines on a seasonal basis to better reflect the diversity present in the population of currently circulating viruses. The method described herein utilizes recent seasonal surveillance information, in combination with novel consensus-based sequence building approaches to provide a unique method for updating broadly reactive influenza virus vaccines designs to better protect people against co-circulating viruses.

In an effort to improve upon the COBRA methodology and enhance vaccine coverage within current and into future influenza A(H3N2) virus seasons, nine next-generation H3 HA antigens were designed utilizing two different scenarios of multi-consensus layering sequence alignment techniques that are unique to this method. These nine next-generation H3 COBRA HA antigens were expressed on the surface of virus like particles (VLP) and used to vaccinate immunologically naïve mice in a prime, boost, boost regimen . Serum samples collected from vaccinated mice were tested against a panel of historical and co-circulating A(H3N2) influenza viruses in both hemagglutination inhibition (HAI) and neutralization assays. Seven of the nine next-generation H3 COBRA HA vaccine candidates outperformed wild-type H3 HA antigens from historical influenza vaccine strains, and displayed the ability to elicit protective HAI antibody titers against numerous co-circulating and future drifted influenza virus variants. The next-generation H3 COBRA HA vaccine antigens also elicited high neutralizing antibody titers against A(H3N2) influenza viruses across a timeframe where 4 different wild-type A(H3N2) vaccine strains were selected as seasonal influenza vaccine candidates. Overall, the H3 HA vaccine antigens produced utilizing this next-generation COBRA methodology were effective at eliciting protective antibody titers against historical, co-circulating, and future drifted strains of A(H3N2) influenza viruses.

## Results

### Phylogenetic characterization of H3 COBRA HAs

**Figure 1:**
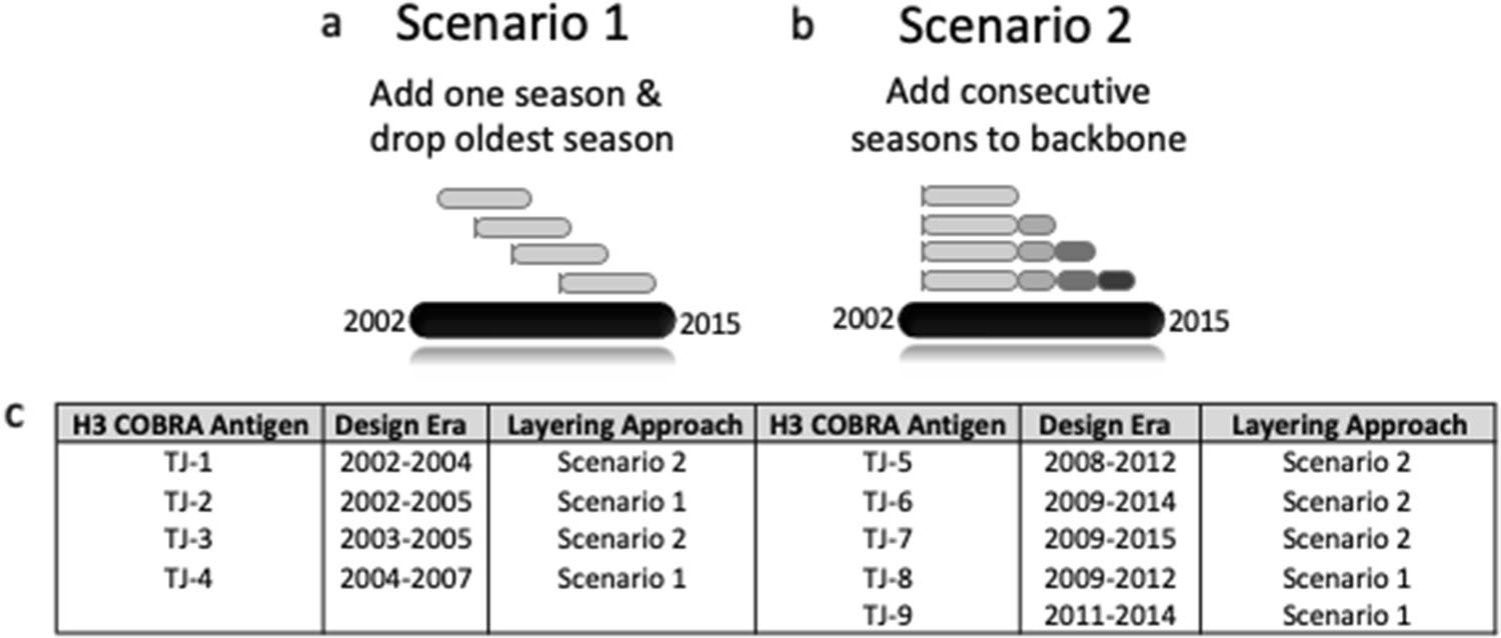
Next-generation H3 COBRA HA multi-consensus sequence layering approach scenarios and final antigen designs. Secondary layer consensus sequences derived from influenza isolates spanning a 2.5 year period are used to generate a “backbone” consensus sequence (light grey bar). (**a**) Scenario 1. Secondary consensus sequences produced from the most recent 6-months of A(H3N2) influenza circulation are added into the backbone sequences, and secondary sequences from the oldest 6-month season of the 3-year period are dropped off. This process is repeated for each 6-month period of the design era. (**b**) Scenario 2. Secondary sequences produced from the most recent 6-months of influenza circulation are added to the “backbone” sequence and the oldest sequences from the design era are retained. This process is repeated for each 6-month period of the design era. (**c**) Description of the next-generation COBRA H3 HA vaccine antigens, their design eras, and the scenario used to generate them. TJ1–4 were designed utilizing wild-type influenza virus HA sequences from 2002 to 2007, and TJ5–9 were generated from HA input sequences that were in circulation from 2008 to 2015.

The next-generation COBRA multilayered consensus building approach was applied to 22,144 human A(H3N2) HA viral amino acid sequences collected from January 1, 2002 to December 31, 2015, that resulted in the generation of 9 unique Next Gen HA sequences. These Next Gen HA antigens were designed to cover influenza A(H3N2) viral isolates over multiple influenza seasons. All next-generation H3 HA amino acid sequences were unique and did not match the amino acid sequence of any HA in a wild-type A(H3N2) isolate. The four HA antigens named TJ-1 to TJ-4 were uniquely designed using input H3N2 HA sequences from 2002 to 2007 and TJ-5 to TJ-9 HA antigens were designed using HA sequences from 2008 to 2015 (Fig. 1c). The antigens were divided into 2 groups because an antigenic shift occurred in H3N2 viruses around 2008 with the emergence of the Perth/09 antigenic cluster^32^.

**Figure 2:**
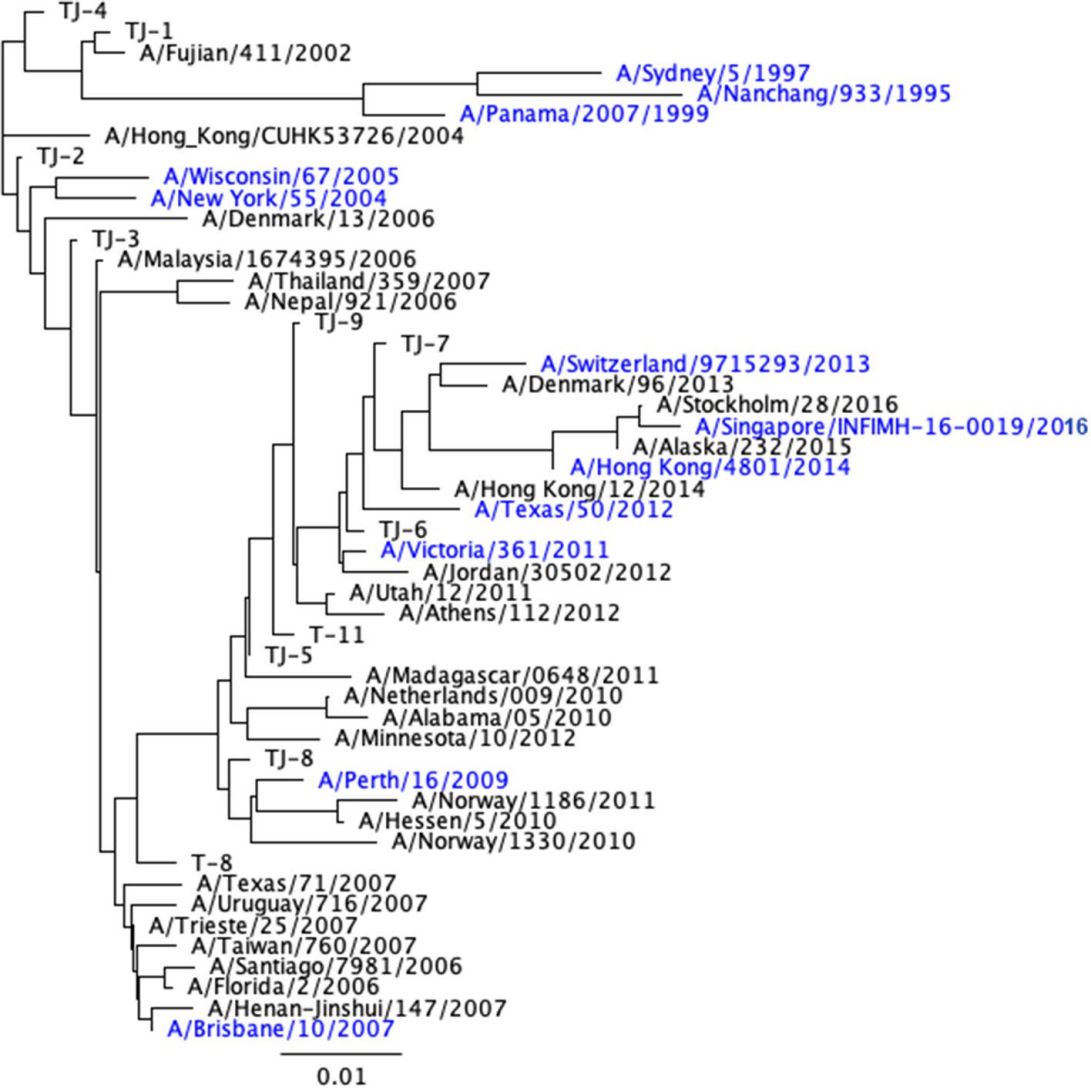
Phylogenetic tree of next-generation COBRA and wild-type H3 HA antigens. The rooted (A/Nanchang/933/1995) phylogenetic tree was inferred from next-generation H3 HA and wild-type H3 HA amino acid sequences derived from representative A(H3N2) influenza viruses isolated from 1995 to 2016 that were obtained from the GISAID EpiFlu online database. Sequences were aligned with MUSCLE 3.8.425 software, and the alignment was refined by Gblocks0.91b software. Phylogeny was determined using a Jukes-Cantor genetic distance model and a neighbor-joining tree building method using Geneious bioinformatics software. Trees were rendered using Geneious tree builder software^55^. Wild-type vaccine strains are highlighted in blue.

Phylogenetic analysis was performed by constructing a phylogenetic tree model utilizing a neighbor-joining tree building method through Geneious Bioinformatics software. The tree was rooted to the ancestral HA amino acid sequence of A/Nanchang/933/1995 A(H3N2) to determine the genetic distance between the designed Next Gen H3 HA proteins and wild-type H3 HA vaccine strains, as well as co-circulating variant HA antigens (Fig. 2).

The TJ-1 to TJ-4 HA amino acid sequences phylogenetically cluster around HA sequences from wild-type viruses isolated between 2002 and 2007. This was expected, as the HA sequence of TJ-1 is most similar to the sequence of the A/Fujian/411/2002 strain and they differ by only two amino acids, V239I and I377T respectively. The HA sequence for TJ-2 is most similar to A/New York/55/2004, with four amino acid differences, A154S, G202V, S235Y, and A546V. TJ-3 is most similar to A/Malaysia/1674395/2006, but carries two amino acid differences at N391D and R466K. TJ-4 is most similar to A/Fujian/411/2002 with four amino acid differences F175Y, N205S, V239I, and P243S (Fig. 2).

As expected, based on their input sequences the TJ-5, 6, 7, 8, and 9 HA sequences are closely aligned with HA sequences from viruses isolated between 2009 and 2014 (Fig. 2). TJ-5 is most similar to A/Madagascar/0648/2011 with five amino acid differences: L13R, I50V, T144A, K427R, and F550S respectively. TJ-6 is most similar to A/Victoria/361/2011, but with 2 amino acid differences at Y9H and S161N. TJ-7 is most similar to A/Hong Kong/12/2014 with 4 amino acid differences, Q49R, Y110H, S328N, and D505N. TJ-8 is most similar to A/Perth/16/2009 with 4 amino acid differences, N160K, H199L, I230S, and S328N. TJ-9 is most similar to A/Athens/112/2012 with 4 amino acid differences, I4V, S70R, N161S, and D503N (Fig. 2). The two traditional COBRA antigens fall in areas of the phylogenetic tree that are similar to the sequences used to design each antigen, with T8 clustering near wild-type viruses from 2009 to 2011, and T11 falling near wild-type viruses from 2010 to 2012 (Fig. 2).

### Antigenic characterization of TJ-1 to TJ-4 H3 COBRA HA proteins

In order to assess the immunogenicity of the next-generation H3 COBRA vaccine antigens, BALB/c mice were vaccinated in a prime, boost, boost regimen with VLPs expressing the first set of next-generation H3 HA proteins, TJ-1 to TJ-4, that were designed for the 2002–2007 era, or VLPs expressing the wild-type H3 HA proteins representing historical vaccine strains from 2002 to 2007. Antisera was collected 14 days after the third vaccination and then tested against a panel of viruses representing A(H3N2) vaccine strains from 1995 to 2016 for HAI activity. Serum from mice vaccinated with TJ-1 VLPs elicited antibodies with HAI titers ≥ 1:40 against 3/12 H3N2 viruses in the panel (the 1997, 1999, and 2002 viruses) (Table 1). TJ-2 and TJ-3 VLP vaccines also elicited antibodies with HAI activity against 3/12 strains (the 2004, 2005, and 2007 viruses). Mice vaccinated with VLPs expressing the TJ-4 or Fuj/02 HA protein had antibodies with HAI activity against the 1999 and 2002 viruses, and vaccination with Wisc/05 or Bris/07 VLPs only elicited antibodies with HAI activity against the homologously matched virus. The mock vaccinated animals did not have antibodies with HAI activity against any of the strains in the panel (Table 1). The COBRA antigens TJ-2 and TJ-3 that were designed for the 2002–2007 era elicited HAI titers ≥ 1:40 against multiple A(H3N2) vaccine strains from the same era that they were designed for while the wild-type H3 vaccines from this era, Wisc/05 and Bris/07, only elicited HAI antibodies ≥ 1:40 against the homologously matched historical vaccine strains. Of note, the TJ-2 and TJ-3 vaccines elicited HAI antibodies against the historical influenza vaccine strains from 2005 and 2007 at titers higher than the homologously matched wild-type vaccine strains.

**Table 1:**
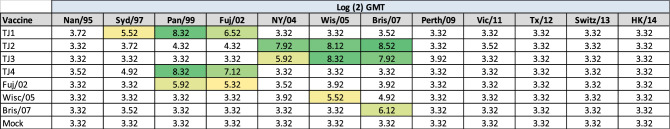
Historical influenza virus HAI panel.

HAI assays were performed against a panel of historical influenza virus vaccine strains isolated from 1995 – 2016 with day 70 serum from vaccinated mice. The average log_2_ geometric mean HAI serum antibody titers (GMTs) were determined for each group of mice (n = 5) and are presented in a heat map form. Colored cells represent groups that achieved a GMT ≥ 5.32, which correlates to an average antibody titer ≥ 1:40. Values closest to 5.32 are colored yellow and become a darker shade of green as the GMT value increases. Cells with no color represent groups that did not achieve a GMT ≥ 5.32.

Sera collected from vaccinated mice was then tested for HAI activity against a panel of viruses representing co-circulating H3N2 influenza strains isolated between 2004 and 2007 (Table 2). Surprisingly, TJ-1 and TJ-4 VLPs were unable to elicit antibodies with HAI activity (≥ 1:40) against any of the viruses in the panel (Table 2). In contrast, mice vaccinated with TJ-3 VLPs had antibodies with HAI activity against 10 of the 12 strains in the panel and mice vaccinated with TJ-2 VLPs had antibodies with HAI activity against all 13 drift variant viruses (Table 2). This was superior to any of the wild-type vaccines tested from this era, of which the best ones, Wisc/05 and Bris/07, elicited HAI antibodies against at most 3/12 strains in the panel at a titer ≥ 1:40. In contrast, the Fuj/02 VLP vaccines were unable to generate antibodies with HAI activity against any of the strains in the panel (Table 2). Mice vaccinated with Wisc/05 VLPs elicited antibodies with seroprotective HAI titers against 3 out of 12 viruses (Malaysia/06, Thailand/07, and Trieste/07). Bris/07 VLPs also elicited seroprotective HAI antibody titers against the Thailand/07 and Trieste/07 strains. Mock vaccinated animals did not have detectable HAI activity against any of the strains in the 2004–2007 co-circulating strains panel (Table 2).

**Table 2:**
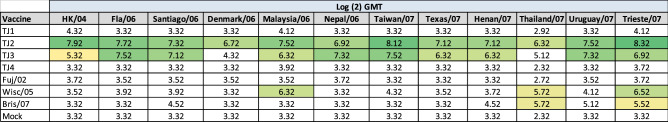
2004–2007 H3N2 Co-circulating influenza virus HAI panel.

HAI assays were performed against a panel of co-circulating influenza strains isolated from 2004–2007 with day 70 serum from vaccinated mice. The average log_2_ geometric mean HAI serum antibody titers (GMTs) were determined for each group of mice (n = 5) and are presented in a heat map form. Colored cells represent groups that achieved a GMT ≥ 5.32, which correlates to an average antibody titer ≥ 1:40. Values closest to 5.32 are colored yellow and become a darker shade of green as the GMT value increases. Cells with no color represent groups that did not achieve a GMT ≥ 5.32.

### Antigenic characterization of TJ 5–9 H3 COBRA HAs

The next-generation COBRA design methodology was then used to create a second round of H3 next-generation HA antigens designed to focus on the 2009–2015 time-frame. These constructs, TJ-5 to TJ-9, were compared to previously designed H3 COBRA VLPs and wild-type H3 VLPs that represented vaccine strains from 2009–2016 against a panel of historical A(H3N2) influenza virus vaccine strains from 1995–2019 for their ability to induce protective levels of HAI reactive antibodies (Table 3). Mice vaccinated with TJ-5 possessed serum with antibodies with HAI activity ≥ 1:40, seroconversion, against all 11 historical vaccine strains isolated from 2005–2019 at levels as high or higher than homologously matched wild-type VLP vaccines (Table 3). Additionally, TJ-5 was the only vaccine to induce seroconversion against the Kan/17 virus, although titers ≥ 1:40 were only achieved by 2 of the 5 animals in the group (data not shown). Vaccination of naïve mice with TJ-6, TJ-7, TJ-8, or Tx/12 induced seroconversion against 8 of the 16 strains in the panel including strains from 2005, 2009–2016, and 2019. TJ-6 also elicited seroprotective antibodies against the Switz/17 strain. TJ-9 and Vic/11 VLP vaccines generated antibodies with seroprotective HAI activity against all of the viruses from 2009–2016, and the SA/19 isolate. Mice vaccinated with T8 COBRA VLP’s generated sero-protective antibodies against H3N2 vaccine strains from 2004 to 2012, and mice vaccinated with T11 seroconverted against H3N2 vaccine strains from 2004 to 2014 (Table 3). The Perth/09 wild-type VLP vaccine caused the mice to seroconvert to the vaccine strains from 2009 to 2016 with the exception of the Switz/13 vaccine strain. Mice vaccinated with Switz/13 VLPs only produced antibodies with HAI activity against viruses from 2011–2013, while mice vaccinated with HK/14 seroconverted against the isolates from 2013–2016 and the SA/19 virus. The Sing/16 VLP vaccines elicited sero-protective antibodies against all of the strains from 2009–2019 with the exception of the Switz/13 and Kan/17 isolates, both of which belong to the co-circulating clade 3c.3a. Mock vaccinated animals did not have HAI activity ≥ 1:40 against any of the strains in the panel (Table 3).

**Table 3:**
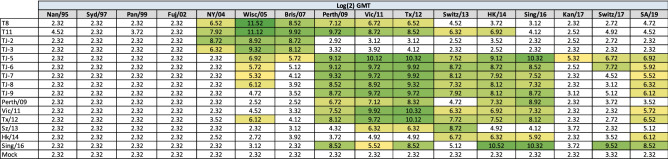
Historical H3N2 influenza virus HAI panel.

HAI assays were performed against a panel of historical influenza virus vaccine strains isolated from 1995 – 2016 with day 70 serum from vaccinated mice. The average log_2_ geometric mean HAI serum antibody titers (GMTs) were determined for each group of mice (n = 5) and are presented in a heat map form. Colored cells represent groups that achieved a GMT ≥ 5.32, which correlates to an average antibody titer ≥ 1:40. Values closest to 5.32 are colored yellow and become a darker shade of green as the GMT value increases. Cells with no color represent groups that did not achieve a GMT ≥ 5.32.

Sera collected from the vaccinated mice was then evaluated against a panel of co-circulating H3N2 strains representing 2010–2016 (Table 4). Mice vaccinated with Perth/09 possessed HAI activity against all of the strains in the panel isolated from 2010 to 2014. All of the next-generation H3 COBRAs, TJ-5 to TJ-9, VLP vaccines elicited similar HAI responses to Vic/11, Tx/12, Switz/13, and HK/14 generating HAI titers ≥ 1:40 against all of the strains in the panel from 2010 to 2016. However, mice vaccinated with TJ-5 had exceptional antibody titers that were as high or higher than the best wild-type vaccines across the panel of co-circulating strains. Mice vaccinated with Sing/16 VLPs or mock vaccines were unable to elicit antibodies with HAI activity ≥ 1:40 against any of the strains in the panel (Table 4).

**Table 4:**
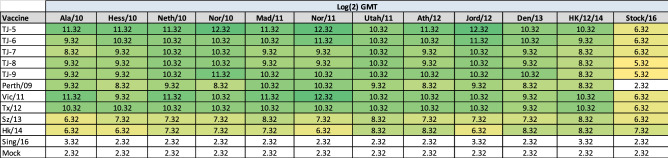
2010–2016 Co-circulating H3N2 influenza virus HAI panel.

HAI assays were performed against a panel of co-circulating influenza strains isolated from 2010–2016 with day 70 serum from vaccinated mice. The average log_2_ geometric mean HAI serum antibody titers (GMTs) were determined for each group of mice (n = 5) and are presented in a heat map form. Colored cells represent groups that achieved a GMT ≥ 5.32, which correlates to an average antibody titer ≥ 1:40. Values closest to 5.32 are colored yellow and become a darker shade of green as the GMT value increases. Cells with no color represent groups that did not achieve a GMT ≥ 5.32.

### H3N2 neutralization assessment

**Figure 3:**
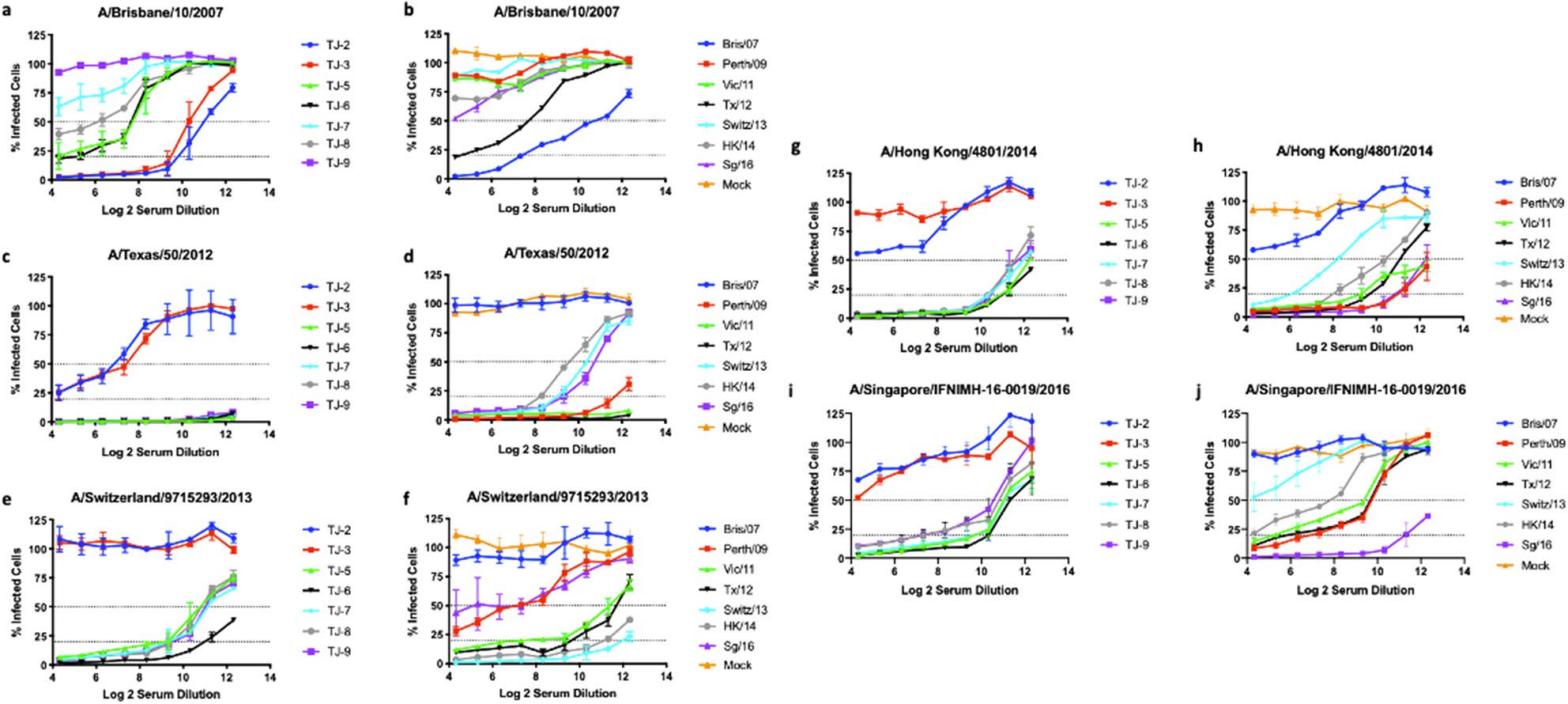
H3N2 focal reduction assay. FRA neutralizing antibody titers were determined using pooled sera for each group of naïve mice vaccinated 3 times with one of 7 next-generation COBRA H3 HA antigens (TJ-2, TJ-3, or TJ5–9) or H3 VLP vaccines expressing wild-type HA proteins (Bris/07, Perth/09, Vic/11, Tx/12, Switz/13, HK/14, or Sing/16) from historical influenza virus isolates at day 70. (**a**,**b**) Average Log_2_ neutralizing antibody titers against the A/Brisbane/10/2007 H3N2 virus. (**c**,**d**) Average Log_2_ neutralizing antibody titers against the A/Texas/50/2012 H3N2 virus. (**e**,**f**) Average Log_2_ neutralizing antibody titers against the A/Switzerland/9715293/2013 H3N2 virus. (**g**,**h**) Average Log_2_ neutralizing antibody titers against the A/Hong Kong/4801/2014 H3N2 virus. (**i**,**j**) Average Log_2_ neutralizing antibody titers against the A/Singapore/IFNIMH-16-2016 H3N2 virus. For each virus, the virus concentration was standardized to 1.2 × 10^4^ FFU/mL. The dotted lines represent the 50% and 80% inhibition of viral infection by antisera compared to virus only control wells.

The ability of vaccine elicited antibodies to neutralize live virus infection was evaluated using a focal reduction assay (FRA) (Fig. 3). Mice vaccinated with TJ-2 or TJ-3 VLPs had the highest antibody neutralization titers amongst all of the next generation COBRA H3 vaccines against the Bris/07 virus, with a FRA_50_ Log(2) titer of 10.38 and 11.06 respectively. Mice vaccinated with TJ-5, TJ-6, or TJ-8 VLP vaccines also elicited a FRA_50_ against Bris/07 albeit at lower titers of 7.71, which was lower than sera from Bris/07 VLP vaccinated mice. Sera from mice vaccinated with TJ-7 and TJ-9 were unable to elicit antibodies that inhibited Bris/07 infection at a level ≥ 50% (Fig. 3a). However, most of the VLP vaccines expressing wild-type HA antigens were also unable to elicit antibodies with neutralization titers against Bris/07, except for the homologous Bris/07 VLP (10.66 FRA_50_ titer) and the Tx/12 VLP (7.79 FRA_50_ titer) (Fig. 3b). Due to their lack of HAI breadth, serum from mice vaccinated with TJ-1 and TJ-4 were not tested in the FRA for their ability to neutralize future emerging strains.

Serum samples collected from mice vaccinated with TJ-2 and TJ-3 VLPs had moderate neutralizing titers against Tx/12 virus, with a FRA_50_ titer of 6.82 and 7.84 respectively. In general, all of the other COBRA HA VLP vaccines, TJ-5 – TJ-9, had antibodies that neutralized the Tx/12 virus at levels greater than 80% in all of the serum dilutions tested (Fig. 3c). The wild-type VLP vaccines Switz/13, HK/14, and Sing/16 all elicited antibodies with moderate capacity to neutralize the Tx/12 virus, while Perth/09, Vic/11, and Tx/12 VLP elicited antibodies that were highly effective at neutralizing the Tx/12 challenge virus. The Bris/07 VLP and mock vaccinated animals did not have antibodies that could prevent Tx/12 infections of the host cells (Fig. 3d).

TJ-5, TJ-7, TJ-8, and TJ-9, vaccines were effective at generating antibodies that inhibit Switz/13 infections with a FRA_50_ of ~ 11.0. Of the Next Gen H3 vaccines, serum collected from mice vaccinated with TJ-6 VLPs were most effective at neutralizing the Switz/13 virus. In contrast, TJ-2 and TJ-3 VLPs failed to neutralize Switz/13 viral infections (Fig. 3e). In general, mice vaccinated with either Vic/11, Tx/12, Switz/13, or HK/14 VLPs were most effective at neutralizing the Switz/13 virus. All of these vaccines neutralized more than 50% of the viral infections at almost all of the dilutions tested, with the homologously matched Switz/13 VLP vaccine eliciting antibodies with the greatest capacity to neutralize the challenge virus. The wild-type vaccines, Bris/07, Perth/09, Sing/16, as well as mock vaccinated animals were much less effective at neutralizing Switz/13 than the other wild-type strains (Fig. 3f).

Mice vaccinated with TJ-2 or TJ-3 VLPs were unable to elicit antibodies that could neutralize the HK/14 virus. However, five COBRA vaccine antigens (TJ-5 to TJ-9) elicited levels of antibodies that were broadly neutralizing across the 2012–2016 timeframe, whereas the wild-type seasonal vaccine antigens from that timeframe elicited antibodies that were more strain specific and less cross reactive (Fig. 3g). Of the wild-type vaccines, Perth/09, Vic/11, Tx/12, HK/14, and Sing/16 VLPs all elicited antibodies that could neutralize the HK/14 with a FRA_50_ greater than 10. The Switz/13 VLP vaccines elicited neutralizing with a FRA_50_ of 8.37, while the Bris/07 VLP and mock vaccines were unable to prevent any virus infections (Fig. 3h).

The TJ-5 to TJ-9 VLP vaccines all elicited antibodies had a FRA_50_ titer of 10.6 or higher against the Sing/16 virus at any serum dilution tested. However, mice vaccinated with either TJ-2 or TJ-3 were unable to neutralize the Sing/16 virus (Fig. 3i). In general, mice vaccinated with wild-type vaccines from 2009 to 2014 had antibodies that prevented more than 50% of the viral infections caused by the Sing/16 virus with an FRA_50_ titer of 9.88. In contrast, the homologously matched Sing/16 VLPs neutralized more than 50% of the infections at all dilutions tested. Switz/13 and Bris/07 VLPs, as well as the mock vaccinated animals, did not elicit antibodies capable of neutralizing the Sing/16 virus (Fig. 3j).

Overall, the next-generation H3 COBRA HA vaccine antigens designed for the 2009–2015 era elicited high levels of neutralizing antibody titers across a timeframe, 2012–2016, where 4 different wild-type A(H3N2) vaccine strains were chosen to be included in the seasonal influenza vaccine. Additionally, the TJ-5 – TJ-9 VLP vaccines elicited antibodies that neutralized infections against the future drifted Sing/16 virus at titers two-fold higher than any wild-type vaccine other than the homologously matched Sing/16 vaccine.

## Discussion

The overall objective of the next-generation COBRA methodology is to apply the usefulness of the in-silico vaccine antigen design process to a more commercial vaccine manufacturing-type setting where vaccines are produced in real-time in response to current antigenic and genetic viral variations. Although vaccines against the influenza virus were developed as early as the 1940′s, the use of inactivated wild-type viruses as vaccines are still the best method of inducing protection in the human population^33^. Current seasonal wild-type A(H3N2) influenza vaccines are typically strain specific and sometimes induce a narrow range of antigenic breadth against co-circulating viral variants^1,30^. However, there are some eras of H3N2 influenza virus circulation where the wild-type vaccines elicit highly cross-reactive antibodies. In this study, the wild-type vaccines that were isolated from 2009 to 2016 elicited cross-reactive antibodies against the strains of this era. This is in contrast to isolates collected prior to this time period that do not elicit cross-reactive antibodies and were often antigenically strain specific^24^. The relatively continuous genetic and antigenic drift of influenza A(H3N2) viruses requires the WHO to perform extensive genetic and antigenic surveillance on circulating isolates, as well as accurately predicting the protective efficacy of candidate vaccine viruses each season^1^. The selection of mismatched strains by the WHO can also lead to greatly reduced vaccine effectiveness, as observed during the 2014–2015 influenza season, where a poor match between the A(H3N2) vaccine strain and those viruses in circulation reduced the vaccine effectiveness to ~ 19% in the U.S.^34^.

There is a need for more broad immune protection against co-circulating A(H3N2) influenza viruses, and improvements need to be made to both vaccine candidate selection and deployment against emerging viral strains^31^. From the moment that vaccine manufacturers receive the strain recommendation from the WHO in late February, there are ~ 6 months to produce and purify enough vaccine to make hundreds of millions of doses^1^. . Speed of production is critical and there would be less pressure if vaccine manufacturers could produce a vaccine that elicits long-lived protective immune responses^33,35^. In 2018, the U.S. government was tasked with expanding the capacity for more agile and rapid vaccine production methods to advance the development of broadly reactive vaccine candidates^30^. The accelerated pace of generating and selecting up to date next-generation COBRA-based antigens would be highly advantageous over traditional methods of selecting wild-type vaccine candidate viruses. Since these COBRA vaccine antigens are designed using the year-round influenza virus surveillance data, a candidate vaccine could be generated in real time, antigenically tested, and selected long before traditional strain recommendations. Therefore, broadly reactive vaccine candidates could be rolled out at any time to combat the antigenic diversity of co-circulating A(H3N2) viral strains.

As a result of a combination of rapid viral mutation, reassortment, natural selection in hosts, and epidemiology, A(H3N2) influenza viruses are evolving in humans faster than any other subtype of influenza virus. The WHO has recommended to change the A(H3N2) influenza strain in the commercial seasonal vaccines 11 times for Northern hemisphere since the 1998–1999 season^31^. The high level of diversity of the surface epitopes present on A(H3N2) influenza viruses are challenging for both immune recognition and traditional vaccine effectiveness. However, the next-generation COBRA HA design approach presented in this report resulted in unique HA sequences that incorporated the antigenic epitopes from numerous co-circulating strains of A(H3N2) influenza viruses transmitting during the most recent influenza virus seasons. This next-generation process reduces potential biases in input sequences by weighting isolates based on sequence identity and time of isolation, thereby allowing the COBRA HA antigens to retain highly immunogenic and cross-reactive epitopes^36^.

Antibodies directed against conserved regions in the HA protein can provide broad protection against many different influenza viruses^37^. It is likely that the next-generation H3 COBRA antigens elicited broadly reactive antibodies in a similar manner to traditional COBRA HA vaccine antigens, by driving diverse B cell responses and targeting conserved HA epitopes that are maintained in future A(H3N2) influenza drift variants. Unlike many other broadly reactive influenza virus vaccine candidates, that direct antibodies to the conserved regions of the HA stem, COBRA HA antigens preferentially direct antibodies against conserved structures of the HA globular head, such as the receptor binding site that remains relatively similar in structure to allow for sialic acid binding functions^36^. This is advantageous since antibodies directed against the HA globular head are induced at higher titers and with greater potency than those directed against the stalk^37,38^. However, whether or not these broadly reactive antibody responses are the result of synergistic polyclonal antibodies targeting multiple conserved epitopes, or are the product of a highly specific monoclonal antibody response targeting one epitope remains unclear. Although, COBRA HA based vaccines have been shown to elicit a broader set of monoclonal antibodies (mAbs) than wild-type HA antigens^36^. Therefore, understanding the mechanism(s) that COBRA HA proteins use to elicit broadly-reactive antibodies is important for the design and improvement of broadly reactive antigens and will be the goal of future investigations.

The methodology presented herein differs from the approach used to generate previous COBRA HA antigens. The next generation strategy places an emphasis on current and recently circulating viruses rather than on historical influenza isolates, antigenic eras, or past outbreak groups. The previously published COBRA methodology is good for producing antigens that are broadly reactive for shorter periods of time, i.e. 5 to 10 year periods, but as wild-type viruses evolve and change, modifications to the broadly reactive vaccine candidate must be made to keep the vaccines up to date. The overall goal of this study was to develop a methodology that would allow manufacturers the ability to keep their broadly reactive vaccine candidates up to date in the event that their current vaccine begins to elicit suboptimal antibody breadth against the viruses in circulation.

Two next-generation COBRA design scenarios were tested in this study for their ability to produce broadly reactive vaccine antigens that elicit antibodies against influenza strains from within and across multiple influenza virus seasons. However, neither scenario proved to be advantageous over the other. In fact, for many of the seasons modeled in this study, both scenarios produced identical antigens (data not shown) further emphasizing that both design scenarios are capable of producing broadly reactive antigens. However, both design approaches also produced antigens, TJ-1 and TJ-4, that were not very broadly reactive. This is likely because the HA sequences of TJ-1 and TJ-4 are most similar to the A/Fujian/411/2002 virus, which belongs to a different antigenic era than the H3N2 influenza viruses in circulation between 2004–2007^24,39^. Both TJ-1 and TJ-4 possess a lysine at HA site 161, which is located in antigenic site B, and a valine at site 242 in antigenic site D, which is identical to the amino acids in those positions for A/Fujian/411/2002. Contrastingly, TJ-2 and TJ-3 possess an asparagine at site 161 and an isoleucine at site 242 which is also carried by the A/Wisconsin/67/2005 strain. Together, these amino acid changes are likely responsible for the observed differences in HAI activity between TJ-1/TJ-4 and TJ-2/TJ-3, especially the differences at site 161 as mutations in antigenic site B are often immunodominant and responsible for antigenic drift^40^. Future studies will focus on deconvoluting the differences between the quality of antigens produced by each of the design scenarios, and their ability to induce antibody breadth against both dominant and co-circulating influenza virus strains within and across multiple influenza seasons.

Overall, the H3 COBRA HA antigens designed using both of the next-generation methodologies were able to broadly elicit both HAI reactive and neutralizing antibodies against historical, co-circulating, and future drifted strains of A(H3N2) influenza viruses at a level that was equivalent or greater than wild-type antigens from the same era. Two of the vaccine candidates in particular, TJ-2 and TJ-5, elicited the strongest anti-influenza antibody responses against the era for which they were designed, as well as against future A(H3N2) influenza virus strains. Subsequent studies will focus on further characterizing the immune responses generated by these two vaccine candidates in various animal models.

Despite the value of testing potential vaccine candidates in immunologically influenza-naïve animal models, these systems only provide information about how these antigens will stimulate a de novo immune response, but most human adults have previously been exposed to influenza viruses either through infection or vaccination and possess immunological memory that will differentially drive immune responses to subsequent vaccinations^41^. Future investigations will aim to determine if the breadth of response generated by these next-generation H3 COBRA vaccines are the result of the stimulation of a larger number of pre-existing memory B cells leading to a more diverse antibody repertoire than those stimulated by wild-type vaccines. Therefore, evaluation of these vaccine candidates in animals with pre-existing immunity elicited by previous exposure(s) to influenza viruses will provide information on how an individual’s immune history plays a role in generating effective antibody responses following vaccination.

In summary, this next-generation antigen design methodology is an effective and novel approach for designing broadly reactive subtype-specific seasonal A(H3N2) influenza virus antigens in real time. This process allows the natural evolution of influenza viruses to dictate the antigen design process, and places an emphasis on currently drifted strains rather than historical strains or making predictions on emerging strains that may or may not become dominant in circulation. Designing custom influenza HA antigens through this next-generation process is likely to reduce the need for frequently updating vaccine components and increase protection within a season. This would be highly advantageous for commercial vaccine manufacturers since it would decrease the risk of including a mismatched strain in the vaccine, while increasing vaccine effectiveness, and allow year-round vaccine production. This next generation methodology provides a way to update broadly reactive influenza vaccine candidates with the use of in-silico designs and real time surveillance information, rather than relying on annual predictions, which is a necessary step forward in the fight to protect humans against the ever-changing landscape of co-circulating H3N2 influenza viruses.

## Materials and methods

### Antigen construction and synthesis

Full length wild-type influenza A(H3N2) HA protein amino acid sequences, residues 1–566 (starting with Methionine as the first amino acid), from 22,144 human H3N2 virus infections collected from January 1, 2002 to December 31, 2015 were downloaded from the Global Initiative on Sharing Avian Influenza Data (GISAID) EpiFlu online database and organized by their date of collection. Unlike the traditional COBRA antigen design method, where input sequences are separated based upon the year in which they were collected, this next generation methodology separates isolates based on the representative influenza “season” in which they were collected^20,24,26^. In this approach, the downloaded sequences were separated into periods of time representing the “Northern” and “Southern” Hemisphere influenza seasons spanning from 2002 to 2015. The “Southern Hemisphere” season was defined to include sequences from all over the globe isolated from 5/1/XX to 9/30/XX of a given year, and each “Northern Hemisphere” season was defined to include sequences from around the world that were isolated from 10/1/XX to 4/30/XY of the following year. For example, sequences included in the 2009 “Southern Hemisphere” collection timeframe included those isolated between 5/1/2009 and 9/30/2009, and the isolates included in the 2009–2010 “Northern Hemisphere” collection contains sequences that were isolated between 10/1/2009 and 4/30/2010, regardless of where the sequences were isolated geographically. The HA amino acid sequences for each season were then aligned using Geneious bioinformatics software (Biomatters, Ltd. Auckland, New Zeland), and separated into multiple distinct clusters, which were then used to generate “primary” consensus sequences. The primary sequence clusters were derived using a 99% identity cutoff, which correlates to approximately 5 amino acid differences in the HA molecule. This cutoff was chosen based on the suggestion that influenza virus strains with more than 4 amino acid differences in their HA proteins are of epidemiological importance^34,42^. This approach differs from the traditional influenza subtype specific COBRA antigen design approach that builds the primary level of consensus sequences from either outbreak groups, antigenic eras, or historical isolates, rather than from the most recent seasons of influenza circulation^20,24,26^.

For each round of primary consensus generation, a multiple-sequence alignment was performed using the Geneious MUSCLE 3.8.425 alignment algorithm, and phylogenetic trees were constructed using the Jukes-Cantor genetic distance model and a neighbor-joining tree building method, such that trees were rooted to the oldest sequences collected. The full-length HA sequences were then aligned and clustered based on 99% identity. Then the most common amino acid residues found among each designated cluster of viruses was used to generate a primary consensus sequence. The multiple primary consensus sequences from each 6-month season were then aligned, phylogenetically analyzed using the previously described method, and clustered into groups based on phylogenetic similarity from which “secondary” level consensus amino acid sequences were generated. The secondary level sequences were then input into 2 different COBRA consensus building scenarios, which is unique to this next-generation COBRA methodology (Fig. 1). Both scenarios start by collecting the secondary sequences from 2.5 years, representing 5 consecutive influenza seasons, aligning them, and using those generate a “backbone” consensus sequence. The first scenario moves forward with time in such a way that the secondary sequences derived from the most recent 6-month period are added to the backbone and the secondary sequences from the oldest season of the now 3-year period are removed (Fig. 1a). This process creates an antigen design window that marches forward with time, placing an emphasis on newly emerging strains and eliminating contributions from older strains. The second COBRA consensus building scenario starts with the same 2.5-year back bone and incorporates secondary sequences from the most recent 6-months of influenza virus circulation. This scenario differs from the first, in that it retains the secondary sequences from the oldest influenza season and also adds in the secondary consensus sequences from the most recent 6-month time period (Fig. 1b). This scenario places value on past sequences while also incorporating sequences from the most recent influenza virus season.

Multiple rounds of consensus assembly were layered in both scenarios to yield secondary, tertiary, and quaternary consensus sequences that were designed to represent different antigenic spaces that overlapped periods of time for which multiple wild-type vaccine strains were recommended for inclusion in seasonal vaccine formulations from 2009 to 2015. The antigens were deliberately designed up to 2015 so that these antigens could be tested as vaccines against future emerging H3N2 isolates. This process generated a combination of 27 COBRA antigen sequences from both scenarios of which 9 were unique. The final set of nine amino acid sequences were named the TJ-series. The nine H3 COBRA HA antigens, TJ-1 through TJ-9, were then synthesized and inserted into the pTR600 plasmid expression vector, as previously described and generated as VLPs^43^. The H3 next-generation HA antigens were designed to represent discrete time periods of recent A(H3N2) influenza virus circulation in humans. For example, as a proof of concept, constructs TJ-1 through TJ-4 were designed to represent past periods of time spanning 5 years, constituting the A(H3N2) influenza antigenic space from 2002 to 2007. This timeframe was chosen such that the antibodies elicited by these antigens could be tested against future A(H3N2) influenza isolates. In a similar manner TJ-5 through TJ-9 were generated using the two multi-consensus layering approaches to represent the time period of 2009–2015 (Fig. 1c).

### Vaccine preparation

Mammalian 293 T cells were transfected with each of three plasmids expressing either the influenza neuraminidase (A/mallard/Alberta/24/2001, H7N3), the HIV p55 Gag sequence, and one of the various influenza A(H3N2) wild-type HA or H3 COBRA HA expressing plasmids in previously described mammalian expression vectors^44^. A(H3N2) wild-type influenza HA sequences were obtained from the GISAID EpiFlu database using MDCK passaged sequences which were inserted into the pTR600 expression vector^21^. This included the HA sequences for following H3 antigens: A/Fujian/411/2002 (EPI_ISL_11184) MDCK passage 1 (MDCKP1), A/Wisconsin/67/2005 (EPI_ISL_115646) MDCKP2, A/Brisbane/10/2007 (EPI_ISL_110723) MDCKP1, A/Perth/16/2009 (EPI_ISL_87516) MDCKP2, A/Victoria/361/2011 (EPI_ISL_121879) MDCKP2, A/Texas/50/2012 (EPI_ISL_170149) MDCKP1, A/Switzerland/9715293/2013 (EPI_ISL_166310) MDCKP2, A/Hong Kong/4801/2014 (EPI_ISL_198222) MDCKP2, A/Singapore/IFNIMH-16-0019/2016 (EPI_ISL_296168) MDCKP1. Additionally, the T8 and T11 COBRA HA sequences, which were generated using the traditional H3 COBRA methodology were inserted into the pTR600 expression vector and synthesized as VLP’s to serve as comparators between the traditional methodology and the newly proposed next generation methodology. T8 was designed using H3 wild-type sequences from 1999 to 2012, and T11 using H3 sequences from 2011 to 2013^24^. Following 72 h of incubation at 37 °C, supernatants from transiently transfected HEK 293-T cells were collected, centrifuged to remove cellular debris, and filtered through a 0.22 μm pore membrane. Mammalian virus-like particles (VLPs) were purified and sedimented by ultracentrifugation on a 20% glycerol cushion at 135,000 × *g* for 4 h at 4 °C. VLPs were resuspended in phosphate buffered saline (PBS) and total protein concentration was assessed by conventional bicinchoninic acid assay (BCA)^19^. Hemagglutination activity of each preparation of VLPs was determined by adding equal volume of 0.75% guinea pig red blood cells (RBCs) to a V-bottom 96-well plate and incubating with serially diluted volumes of VLPs for a 60 min incubation at room temperature (RT). The highest dilution of VLP with full agglutination of RBCs was considered the endpoint HA titer.

### Determination of HA content

A high-affinity, 96-well flat bottom ELISA plate, Immulon 4 HBX (Thermo Fischer, Waltham, MA, USA), was coated with 5–10 μg of total protein of VLP and serial dilutions of a recombinant H3 antigen (3006_H3_Vc, Protein Sciences, Meriden, CT) in ELISA carbonate buffer (50 mM carbonate buffer, pH 9.5) and the plate was incubated overnight at 4 °C on a rocker. The next morning, plates were washed in PBS with 0.05% Tween-20 (PBST), then non-specific epitopes were blocked with 1% bovine serum albumin (BSA) in PBST solution for 1 h at RT. Buffer was removed then stalk-specific Group 2 human antibody CR8020 (Sanofi Pasteur, Lyon, France) 1 mg/mL, was added to the plate in blocking buffer at a working dilution of 1:4000, and incubated for 1 h at 37 °C^45^. Plates were washed, then probed with goat anti-human IgG horseradish-peroxidase-conjugated secondary antibody (1 mg/mL) diluted in blocking buffer at a working dilution of 1:4000 (2040-05, Southern Biotech, Birmingham, AL, USA) for 1 h at 37 °C. Plates were washed then freshly prepared o-phenylenediamine dihydrochloride (OPD) (P8287, Sigma, St. Louis, MO, USA) substrate in citrate buffer (P4922, Sigma, St. Louis, MO, USA) was added to wells for 3–5 min, followed by 1 N H_2_SO_4_ stopping reagent. Plates were read at 492 nm absorbance using a microplate reader (Powerwave XS, Biotek, Winooski, VT, USA) and background was subtracted from negative wells. Linear regression standard curve analysis was performed using the known concentrations of recombinant standard antigen to estimate HA content in VLP lots^19^. COBRA constructs were also analyzed for glycan content by searching for potential glycosylation motifs in the amino acid sequence. Using the sequence motif, N{P}[ST]{P}, to search for potential N-linked glycosylation sites using Geneious annotation software, it was determined that TJ5 through TJ9 all possess the same 12 potential glycosylation sites, and these sites are consistent with those found in wild-type HA’s isolated in the last 10 years.

### Phylogenetic comparison of next-generation COBRA H3 HA antigens

A rooted (A/Nanchang/933/1995) phylogenetic tree was inferred from next-generation H3 HA and wild-type HA amino acid sequences derived from representative H3N2 viruses isolated from 1995 to 2016. Sequences were aligned with MUSCLE 3.8.425 software, and the alignment was refined by Gblocks0.91b software. Phylogeny was determined using a Jukes-Cantor genetic distance model and a neighbor-joining tree building method with Geneious bioinformatics software. Trees were rendered using Geneious tree builder software (Biomatters, Ltd. Auckland, New Zeland) (Fig. 2).

### Viruses and HA antigens

Influenza A(H3N2) viruses were obtained through the Influenza Reagents Resource (IRR), BEI Resources, the Centers for Disease Control (CDC), or provided by Virapur (San Diego, CA). Viruses were passaged once in the same growth conditions as they were received, in either embryonated chicken eggs or semi-confluent Madin-Darby canine kidney (MDCK) cell culture as per the instructions provided by the WHO^46^. Virus lots were titered with 0.75% guinea pig erythrocytes in the presence of 20 nM Oseltamivir, and made into aliquots for single-use applications.

The A(H3N2) 1995–2019 WHO recommended historical influenza vaccine strain viral panel included the 16 following viral strains: A/Nanchang/933/1995 (Nan/95) egg passage 3 (EP3), A/Sydney/05/1997 (Syd/97) EP2, A/Panama/2007/1999 (Pan/99) EP4, A/Fujian/411/2002 (Fuj/02) MDCKP1, A/New York/55/2004 (NY/04) EP6, A/Wisconsin/67/2005 (Wisc/05) EP4, A/Brisbane/10/2007 (Bris/07) EP3, A/Perth/16/2009 (Per/09) EP4, A/Victoria/361/2011 (Vic/11) EP4, A/Texas/50/2012 (Tx/12) EP4, A/Switzerland/9715293/2013 (Switz/13) EP4, A/Hong Kong/4801/2014 (HK/14) EP11, and A/Singapore/IFNIMH-16-0019/2016 (Sing/16) EP3, A/Kansas/14/2017 (Kan/17) EP1, A/Switzerland/8060/2017 (Switz/17) EP1, A/South Australia/34/2019 (SA/19) EP1.

The panel of 24 co-circulating H3N2 viral variants from the period of 2004–2016 included:, A/Florida/02/2006 (Fla/06) EP2, A/Santiago/7981/2006 (Santiago/06) EP1, A/Nepal/921/2006 (Nepal/06) EP1, A/Taiwan/760/2007 (Taiwan/07) MDCKP1, A/Texas/71/2007 (Texas/07) MDCKP1, A/Henan-Jinshui/147/2007 (Henan/07) EP1, A/Uruguay/716/2007 (Uruguay/07) EP2, A/Alabama/05/2010 (Ala/10) MDCKP2, A/Hessen/5/2010 (Hess/10) MDCKP3, A/Netherlands/009/2010 (Neth/10) MDCKP2, A/Norway/1330/2010 (Nor/10) MDCKP3, A/Madagascar/0648/2011 (Mad/11) MDCKP2, A/Norway/1186/2011 (Nor/11) MDCKP2, A/Utah/12/2011 (Utah/11) MDCKP2, A/Athens/112/2012 (Ath/12) MDCKP2, A/Jordan/30502/2012 (Jord/12) MDCKP2, A/Denmark/96/2013 (Den/13) MDCKP2, A/Hong Kong/12/2014 (HK/12/14) MDCKP2, A/Stockholm/28/2016 (Stock/16) MDCKP2.

When viruses could not be obtained, H3-A/Thailand/1/2004 N1-Gag VLPs were synthesized using codon optimized HA sequences. This included the following antigens for the 2004–2007 drift panel: A/Hong Kong/CUHK53726/2004 (HK/04) (EPI_ISL_14373) MDCKP1, A/Denmark/13/2006 (Denmark/06) (EPI_ISL_16064) MDCKP1, A/Malaysia/1674395/2006 (Malaysia/06) (EPI_ISL_120397) MDCKP1, A/Thailand/359/2007 (Thailand/07) (EPI_ISL_20528) MDCKP1, and A/Trieste/25/2007 (Trieste/07) (EPI_ISL_98951) MDCKP1.

### Phylogenetic comparison of co-circulating variants (2004–2016)

Selection of the co-circulating variants was based upon multiple sequence alignments that were performed on extracted HA sequences; sequences were organized based on collection during the representative “Northern” or “Southern” Hemisphere influenza seasons from 2004 to 2016. Phylogenetic tree models were assembled using a Jukes-Cantor genetic distance model, and a neighbor-joining tree building method in Geneious bioinformatics software (Biomatters, Ltd. Auckland, New Zeland). Branches were compared for sequence similarity to the panel of 24 drift viruses described above. The number of sequences that branched within 99% HA sequence identity of one of the 24 antigens was counted, and frequencies were calculated based on the total number of sequences available per season as described previously^19^.

### VLP Vaccination of mice

BALB/c mice (*Mus musculus*, female 6–8 weeks old) were purchased from the Jackson Laboratory (Bar Harbor, ME, USA), housed in microisolator units, and allowed free access to food and water. They were cared for under the University of Georgia Research Animal Resources guidelines for laboratory animals. All procedures were reviewed and approved by the Institutional Animal Care and Use Committee (IACUC). Mice (5 per group) were vaccinated with purified H3 VLPs (3.0 ug HA/mouse/vaccination) based upon HA content from the ELISA quantification, and vaccines were delivered via intramuscular injection at week 0 (3ug) and then boosted with the same vaccine antigen and dose on weeks 4 (3ug) and 8 (3ug), as performed in previous studies^24^. Vaccines were formulated with an emulsified squalene-based oil-in-water emulsion adjuvant, Addavax (InvivoGen, San Diego, CA, USA). The final concentration after mixing 1:1 with VLPs is 2.5% squalene. Mice were vaccinated with either one COBRA H3 (TJ-1 through TJ-9) VLP vaccines or one wild-type (Fuj/02, Wisc/05, Bris/07, Perth/09, Vic/11, Tx/12, Switz/13, HK/14, Sing/16) H3 VLP vaccine with HA antigens representing selected vaccine strains isolated from 2002–2017. Fourteen days after each vaccination, blood samples were collected via the submandibular vein and transferred to a microcentrifuge tube. The tubes were centrifuged at 10,000 rpm for 10 min, and serum samples were removed and frozen at -20 °C ± 5 °C^24^.

### Hemagglutination-inhibition (HAI) assay

The hemagglutination inhibition (HAI) assay was used to assess functional antibodies to the HA that are able to inhibit agglutination of guinea pig erythrocytes. The protocols were adapted from the WHO laboratory influenza surveillance manual^46^. Guinea pig red blood cells are frequently used to characterize contemporary A(H3N2) influenza strains that have developed a preferential binding to alpha (2,6) linked sialic acid receptors^47,48^. To inactivate nonspecific inhibitors, sera samples were treated with receptor-destroying enzyme (RDE) (Denka Seiken, Co., Japan) prior to being tested. Briefly, three parts of RDE was added to one part of sera and incubated overnight at 37 °C. RDE was inactivated by incubation at 56 °C for 30 min. RDE-treated sera were diluted in a series of twofold serial dilutions in in v-bottom microtiter plates. An equal volume of each A(H3N2) influenza virus, adjusted to approximately 8 hemagglutination units (HAU)/50 μl in the presence of 20 nM Oseltamivir carboxylate, was added to each well. The plates were covered and incubated at room temperature for 30 min, and then 0.75% guinea pig erythrocytes (Lampire Biologicals, Pipersville, PA, USA) in PBS were added. Red blood cells were washed with PBS, stored at 4 °C, and used within 24 h of preparation. The plates were mixed by gentle agitation, covered, and the RBCs were allowed to settle for 1 h at room temperature. The HAI titer was determined by the reciprocal dilution of the last well that contained non-agglutinated RBCs. Positive and negative serum controls were also included for each plate. All mice were negative (HAI ≤ 1:10) for pre-existing antibodies to currently circulating human influenza viruses prior to vaccination, and for this study sero-protection was defined as HAI titer > 1:40 and seroconversion as a fourfold increase in titer compared to baseline, as per the WHO and European Committee for Medicinal Products to evaluate influenza vaccines^49^. Mice are naïve and seronegative at the time of vaccination, and thus seroconversion and sero-protection rates are interchangeable in this study.

### Focus reduction assay (FRA)

The Focus Reduction Assay (FRA) used in this study was initially developed by the WHO collaborating Centre in London, U.K. and modified by U.S. Centers for Disease Control and Prevention (CDC) (Thomas Rowe, personal communication). MDCK-SIAT1 cells (Sigma, St. Louis, MO, USA) were plated at 2.5–3 × 10^5^ cells/mL (100 µL/well in 96-well plate) one day prior to use in the assay. Cells were cultured in Dulbecco’s Modified Eagle Medium (DMEM) containing 5% heat-inactivated fetal bovine serum and antibiotics, Geneticin (Gibco, Waltham, MA, USA), in 96-well flat bottom plates overnight to form a 95–100% confluent monolayer. The following day, the cell monolayers are rinsed with 0.01 M phosphate-buffered saline pH 7.2 (PBS, Gibco, Waltham, MA, USA), followed by the addition of twofold serially diluted RDE treated serum (50uL per well) starting with a 1:20 dilution in virus growth medium containing TPCK-treated trypsin (1 µg/ml), VGM-T, (DMEM containing 0.1% BSA, 1% Penicillin/Streptomycin (100 U/mL Penicillin, 100 ug/mL Streptomycin solution), and 1 µg/mL TPCK-treated trypsin (Sigma, St. Louis, MO, USA). 50uL of A(H3N2) influenza virus (1.2 × 10^4^ focus forming units (FFU)/mL, which corresponds to 600 FFU/50 μl) in VGM-T was added to the wells of each plate, or VGM-T only was added to cell control wells. Virus stocks were standardized by previous titration in the FRA^50,51^. Following a 2 h incubation period at 37** °C** with 5% CO_**2**_, the cells in each well were then overlaid with 100uL of equal volumes of 1.2% Avicel RC/CL (Type: RC581 NF; FMC Health and Nutrition, Philadelphia, PA, USA) in 2X Modified Eagle Medium containing 1 µg/mL TPCK-treated trypsin, 0.1% BSA and antibiotics^52^. Plates were incubated for 18–22 h at 37** °C**, 5% CO_2_. The overlays were then removed from each well and the monolayer was washed once with PBS to remove any residual Avicel. The plates were then fixed with ice-cold 4% formalin in PBS for 30 min at 4** °C**, followed by a PBS wash and permeabilization using 0.5% Triton-X-100 in PBS/glycine at room temperature for 20 min. Plates were washed three times with wash buffer (PBS, 0.1% Tween-20; PBST) and then incubated for 1 h with a monoclonal antibody against the influenza A nucleoprotein^51,53,54^ obtained from the Influenza Reagent Resource (IRR) (Manassas, VA, USA) (FR-1217) (1 mg/mL), diluted 1:2000 in ELISA buffer (PBS,10% horse serum, 0.1% Tween-80). Following washing (3X PBST), the cells were incubated with goat anti-mouse peroxidase-labelled IgG (Sera Care, Inc., Milford, MA, USA) (KPL 474–1802) (1 mg/mL), diluted 1:2000 in ELISA buffer for 1 h at RT. Plates were washed again (3X PBST) and infectious foci were visualized using TrueBlue substrate (Sera Care, Inc., Milford, MA USA) containing 0.03% H_**2**_O_**2**_ incubated at room temperature (RT) for 10 min. The reaction was stopped by washing five times with diH_2_0. Plates were air dried and foci enumerated using a CTL BioSpot Analyser with ImmunoCapture 6.4.87 software (CTL, Shaker Heights, OH, USA). The FRA titer was reported as the reciprocal of the highest dilution of serum corresponding to 50% foci reduction compared to the virus control minus the cell control.

In order for a plate to pass quality control, both the average of the octuplet virus control wells (VC), as well as the average of the octuplet cell control wells (CC) must pass. The virus controls initially were between 150 to 650 foci and the cell controls must be less than 21 foci. The virus control wells were subsequently expanded to between 200 and 1600 foci. Additionally, the positive control, A(H3N2) historical influenza vaccine strain, virus was run in triplicate plates in each individual assay and at least two out of three plates must pass VC and CC criteria and homologous ferret antisera, previously generated through infection with A(H3N2) influenza virus at 1e6 PFU/mL and collected 14 days post infection, must have the same titer across the plates^19^. Each assay plate (one virus per plate) contained a panel of ferret reference antisera, as well as a human influenza vaccine serum control to assess overall assay consistency^24^. The percentage of infected cells reported in the assay is calculated by averaging the foci count from the positive control (virus and cell only) wells, and dividing the number of foci in each experimental well by the average of the positive control.

## Data Availability

The data that support the findings of this study are available from the corresponding author upon reasonable request.
